# Exploring perceived costs and benefits of first aid for youth with depression: a qualitative study of Japanese undergraduates

**DOI:** 10.1186/s13033-020-00366-7

**Published:** 2020-05-24

**Authors:** Jun Kashihara, Shinji Sakamoto

**Affiliations:** 1 College of Humanities and Sciences, Nihon University, 3-25-40 Sakurajosui, Setagaya-ku, Tokyo, 156-8550 Japan; 2 Melbourne School of Population and Global Health, The University of Melbourne, 235 Bouverie St, Carlton, VIC 3053 Australia; 3 Japan Society for the Promotion of Science, Kojimachi Business Center Building, 5-3-1 Kojimachi, Chiyoda-ku, Tokyo, 102-0083 Japan; 4 Present Address: Faculty of Sociology, Toyo University, 5-28-20 Hakusan, Bunkyo-ku, Tokyo, 112-8606 Japan

**Keywords:** Depression, Youth Mental Health First Aid, Helping behavior, Cost and benefit, Motivation, Economic perspectives, Qualitative study, Japanese

## Abstract

**Background:**

Early interventions for depression among youth are greatly needed. Although Youth Mental Health First Aid (YMHFA) program has been developed to teach the public how to help young people with mental disorders, including depression, it has assumed human altruism and overlooked the possibility that participants would experience conflict between the costs and benefits of helping behaviors. The present qualitative study, therefore, initially explored content of the costs and benefits perceived by youth in terms of helping their peers with depression.

**Methods:**

A total of 56 Japanese undergraduates (32 female, 24 male; *M*_*age*_ = 20.20, *SD* = 1.09) participated in the face-to-face survey. They were provided with basic knowledge about helping behaviors and were presented with a vignette describing an undergraduate with depression. Then, they left free descriptive comments on their views of the costs/benefits of helping/not helping the person in the vignette. As supplemental quantitative analyses, we statistically compared numbers of labels (*n* = 624), which were obtained from participants’ comments, across two (costs/benefits) × two (helping/not helping) domains. Finally, we conducted a qualitative content analysis that combined inductive and deductive methods to categorize these labels.

**Results:**

The supplemental quantitative analyses (i.e., ANOVA and post hoc analyses) on the numbers of labels highlighted that the participants perceived suppressors (i.e., *costs of helping*, *benefits of not helping*) as well as motivators (i.e., *costs of not helping*) in making decision to help peers with depression. The qualitative content analysis mainly showed that: (i) the categories in each domain covered multiple facets of costs and benefits, including negative/positive effects on the participants themselves, the person in the vignette, and interpersonal relationships; and that (ii) the participants perceived the conflicts of costs and benefits regardless of whether they help their peers with depression.

**Conclusions:**

These results provide evidence for how young people experience the conflicts between the costs and benefits of helping behaviors toward their peers with depression and reveal specific content of these costs and benefits. These findings could serve as a basis for extending YMHFA programs and designing educational content to promote public helping behaviors in realistic situations.

## Background

Depression (i.e., major depressive disorder, as defined in the *Diagnostic and Statistical Manual of Mental Disorders, 5th edition* [DSM-5] [1]) predicts serious societal problems, including suicide [2]. Further, it is estimated to be the largest factor in disease burden by 2030 [3]. Depression affects 350 million people worldwide [4] and 1.1 million people in Japan [5], and prevalence of mental disorders, including depression, peaks during adolescence and early adulthood globally [6–8]. In addition to the high prevalence of depression among youth, research has indicated that mental disorders, including depression, contribute 45% of the disease burden among youth (age 10–24 years) [9], and that early onset of mental disorders has negative influences on many spheres of patients’ later lives, such as educational attainment [10] and marital stability [11].

Early interventions of depression among youth are therefore greatly needed; however, the majority of young people with depression are reluctant to seek professional help. The World Health Organization estimated that less than 30% of people with depression in most countries including Japan receive any professional treatment [12], and similar trends of reluctance to seek help are observed among samples of youth [13, 14]. Research has shown that there are many barriers to help-seeking for youth with depression: people with depression, including youth, often experience public stigma [15–17], and they tend to ignore the positive aspects of helping behaviors (i.e., voluntary actions that are intended to help other individuals) [18, 19] because of cognitive distortions [20]. Moreover, young people tend to lack mental health literacy, which is defined as “knowledge and beliefs about mental disorders which aid their recognition, management or prevention (p. 182)” [21], and are fearful of professional mental health treatments [22–24].

Considering these barriers for youth with depression and the difficulty they face in seeking professional help themselves, it is important to encourage members of the public to recognize young people with depression and teach them how to engage in appropriate helping behaviors. To satisfy the necessity of improving the public’s mental health literacy, Youth Mental Health First Aid (YMHFA) program, targeting those who communicate with young people on a daily basis (e.g., peer students, teachers, parents, sport coaches), has been developed in Australia [25]. To develop the YMHFA program, Kelly et al. [25] added youth-specific content (e.g., knowledge concerning eating disorders and non-suicidal self-injury) to the original version of the Mental Health First Aid (MHFA) program [26–29], which focuses on helping behaviors toward adults with mental disorders, including depression. Similar to the original MHFA, the YMHFA consists of lectures and role plays used to teach five steps of helping behaviors referred to as “ALGEE” to the trainees. These steps are: (A) Assess for risk of suicide or harm, (L) Listen nonjudgmentally, (G) Give reassurance and information, (E) Encourage appropriate professional help, and (E) Encourage self-help and other support strategies. This 14-h program can be conducted as either over two full days or four small sessions, and those instructors registered on the MHFA website [30] teach how to recognize and help young people with mental disorders and facilitate role plays between participants to develop practical skills of helping behavior (for more details, see [25]). An evaluation study of the YMHFA in Australia has demonstrated improvements in knowledge of mental disorders, stigmatizing attitudes, and confidence in offering help [25], and this program has been conducted in the United States [31–33]. To improve literacy regarding the worldwide problem of depression among youth, it is hoped that the YMHFA will be used globally in future, as well as the original MHFA that has already been conducted and evaluated in over 22 countries [34], including the United Kingdom [35, 36], the United States [37], and Japan [38–40].

Evaluation studies of the YMHFA program, however, have indicated that improving mental health literacy is insufficient for increasing appropriate helping behaviors in the real world. Although previous evaluation studies of the YMHFA reported improvements in confidence in offering help [25, 33], understanding of the five steps of helping behaviors [31], and recognizing the importance of offering help [32], they did not show that the programs significantly increased the numbers of actual helping behaviors. Why was the existing YMHFA program not able to effectively increase appropriate helping behaviors? One plausible answer is that these programs have lacked perspectives of motivating participants to engage in helping behaviors. Previous social psychological theories that have explained the determinants of helping behavior [41, 42] have negated the idea of pure altruism. Instead, they have claimed that a cost–benefit analysis, which is an economic way of decision-making focused on minimizing one’s costs and maximizing one’s benefits [43], acts as a key motivator of helping behavior. These psychological theories have argued that people have to estimate the potential results (e.g., the effects on their mental states and their reputation among the community) in advance to decide whether they should engage in the helping behavior [41, 42]. These economic perspectives of helping behavior that emphasize self-interest are supported by various empirical findings. For example, Maner et al. [44] showed that not the participants’ empathy for others but motivation to avoid their own negative affect predicted helping behaviors in daily life situations. Moreover, previous experimental studies have shown that the numbers of helping behaviors in daily life are increased by offering interventions to reduce the perceived costs of helping [45], promoting the recognition of the benefits of helping [46], and promoting the recognition of the costs of not helping [47].

As well as Inagaki and Orehek [48] pointed out in the case of the research on helping behavior in general, the existing YMHFA program have implicitly assumed human altruism and overlooked the conflicts involved in the costs and benefits of providing help. Rather, as illustrated in the conceptual model of mental health literacy [21, 49] used in the YMHFA program, they have emphasized how to improve knowledge of mental disorders, including depression, and helping skills. Although a recent theoretical study on helping behaviors toward people with depression pointed out that perceived costs and benefits act as key determinants of helping behavior among caregivers [50], no empirical studies have explored what kinds of costs and benefits are perceived by the public regarding helping behavior toward people with depression.

To overcome these limitations in the existing YMHFA program, we conducted a qualitative study to explore what kinds of costs and benefits are perceived among a Japanese sample regarding first aid for people with mental disorders. In particular, we examined the perceived costs and benefits regarding helping behavior toward people in early adulthood (i.e., around 20 years old) with depression, considering that early interventions for depression in early adulthood are greatly needed, as reviewed above, and that the strengths and content of public perceptions can differ according to the various types of mental disorders [51–53]. We aimed to initially explore and list the perceived costs and benefits of helping behavior toward youth with depression and to contribute to the existing YMHFA program regarding the economic perspectives of helping behavior. We also expected that the present examination would serve as a theoretical basis for future research with which to design educational content to solve conflicts in the perceived costs and benefits of helping behavior and to motivate the public to conduct first aid for young people with depression.

To conduct an initial exploration of the perceived costs and benefits of first aid for youth with depression, we selectively recruited Japanese undergraduates who majored in psychology and focused on peer support for depression provided on campus for the following four reasons. First, we could access university samples more easily than we could those of high schools, and we intended to obtain a sufficient sample size for the investigation. Second, it is natural for the Japanese to assume that youth with depression are likely to be recognized on campus, because a majority of Japanese young people go to universities and seldom belong to local communities such as sports clubs or religious groups. Third, considering the environment of Japanese universities, in which teaching staff seldom have contact with their students outside of class, we expected that undergraduates would have more chances to recognize peers with depression on campus than would the teaching staff in Japan.

Regarding the content of the perceived costs and benefits of first aid that the present study explored and listed by qualitative analyses, we did not have any a priori hypotheses. Although we also conducted some supplementary quantitative analyses as detailed later, these analyses were not conducted to test specific hypotheses but to describe the characteristics of the present data.

## Methods

### Participants and procedures

We recruited participants from introductory psychology classes held at the College of Humanities and Sciences, Nihon University, which is a middle-ranked private college located in Tokyo. At the beginning of these classes held in the second of two semesters in 2017, we briefly explained the objective and content of the study, the amount of time required for participation, and the content of incentives. We also explained that participation in the study is voluntary and has nothing to do with grades in the classes. Subsequently, we received lists of possible dates from the undergraduates who wished to participate in the study. With the aim of examining beliefs of youth without depression who had basic knowledge of it, we selectively recruited undergraduates who majored in psychology^1^ and reported that they had no history of being diagnosed with clinical depression. A total of 56 Japanese undergraduates (32 female, 24 male; *M*_*age*_ = 20.20, *SD* = 1.09) participated in the survey as a result.

We asked the participants to list possible dates for their participation, divided them into six groups of 6–13 people each, based on the possible dates they listed, and conducted the same survey for each group in a laboratory setting.^2^ All the participants were first informed of the objective and content of the survey in detail and they provided written consent for their participation and journal publication before the investigation. They then completed a brief demographic questionnaire. Next, they watched a slideshow on a screen with voice-over narration, which provided them with basic knowledge of helping behaviors and presented a vignette describing an undergraduate with clinical depression. Finally, they completed a free description sheet to respond with their views on the costs/benefits of helping/not helping the person in the vignette. After they participated, they received incentives (a coupon and an erasable ink pen) worth 700 Japanese yen (i.e., approximately 6.40 U.S. dollars). The protocol of the present study was approved by the Committee of Research Ethics at the Colleges of Humanities and Sciences of the Nihon University (reference number: 29-59).

### Demographic questionnaire

In the demographic questionnaire, we asked participants’ age, gender, nationality, and levels of contact with people with depression. To assess their levels of contact, we used the Japanese-translated version [56] of the Level of Contact Report [57]. This measure is a checklist with 12 statements that reflect different levels of contact with people with depression, ranging from the least (“I have never observed a person that I was aware had major depression”, rank order score = 1) to the most (“I have depression”, rank order score = 12) intimate contact. Participants checked every statement that corresponded to their experience. Each participant’s level of contact was scored by taking the highest rank order score of the statements that (s)he checked.

### Slideshow

The slideshow used in the present study lasted for approximately five minutes and consisted of two parts (for an English-translated version, see Additional file 1). The first part of the slideshow introduced basic knowledge concerning helping behaviors. To create the first part, we referred to social psychological papers [42, 44–46, 48, 50] and books [41, 43, 47] that explained the processes and determinants of helping behavior. In particular, we emphasized that the awareness of someone needing help and empathy is not the only determinant of helping behavior, and that cost–benefit analysis serves as a key determinant. The second part presented the vignette, which is explained in detail in the next subsection. At the end of the second part, based on MHFA guidelines for depression [27, 29, 58, 59], we explained that psychiatrists recommend taking one’s time and listening to friends with depression.

### Vignette

An English-translated version of the vignette used in the present study is available in Additional file 2. To develop the vignette describing an undergraduate with depression, we referred to the Japanese-translated version [60] of the depression vignette used in a national survey of young Australians [61], which was originally written to satisfy the DSM-IV criteria [62]. We then added some visual information presenting the person in the vignette “A” as not doing well (e.g., “A” has dark circles under his/her eyes; “A” has lost weight and looks pale) to increase the ecological validity of the vignette. Furthermore, for the purpose of the present study investigating the perceived costs and benefits of helping a peer with depression, we added the following two modifications. First, at the beginning of the vignette, we explained that “A” is a good university friend of the readers, and that the readers and “A” see each other often both during and outside of classes. Second, we instructed readers to imagine that they recognized “A” as being clinically depressed, and that “A” needs help from someone to deal with his/her problem.^3^

### Free description sheet

The participants were instructed to imagine the situation described in the vignette and to respond with the perceived costs/benefits of helping/not helping “A” using a free description sheet (for an English-translated version, see Additional file 3). We intended to focus on the initial steps of the MHFA guidelines, and defined the term “helping behavior” as taking one’s time and listening to “A.” The sheet was divided into 2 (costs/benefits) × 2 (helping/not helping) domains, and the participants were instructed to write down as many answers as they could in the appropriate domains for 12 min.^4^ With the aim of exploring determinants of helping behaviors other than empathy, we instructed the participants to think about the costs and benefits for themselves, rather than “A.”

### Data analysis

We first summarized participants’ levels of contact with people with depression to clarify the sample characteristics. Next, we summarized the numbers of comments and labels obtained in each domain and conducted a one-way repeated analysis of variance (ANOVA) as a supplemental analysis that statistically compare the numbers of labels across the four domains. We then conducted qualitative content analysis to categorize the free descriptive data. With reference to a guideline of qualitative content analysis that recommended a combination of inductive category development and deductive category application [65], we analyzed free descriptive data in the following stepwise manner. First, the first author of this article divided each comment containing multiple concepts to fit under either two or three labels. Second, the first author and undergraduate staff inductively classified these labels into categories and subcategories. Third, the first and second author examined whether the initial categories and subcategories were plausible by deductively checking the labels, and they elaborated the definitions of the categories and subcategories, which are described later in the Results section. At this step, we assigned codes to the labels indicating the categories and subcategories under which those labels were classified. For example, when we classified a label into the first subcategory in Category 1 in Domain A (see the Results section for the definitions of categories and subcategories), we assigned the code A-1-1 to that label. Fourth, a cooperator who did not know the objective of the present study re-classified the labels independently and deductively. She examined the content of all labels and then assigned one code to each label as we did, with reference to the elaborated definitions of categories and subcategories. Fifth, we compared the codes (i.e., classifications) recorded at steps 3 and 4 and calculated Cohen’s κ coefficients [66] between them to examine the inter-rater reliability of the categories and subcategories we developed. Sixth, and finally, the first author and the cooperator discussed why some disagreements occurred in their analyses and finalized the classifications for each label. To summarize the participants’ levels of contact with people with depression and calculate the resulting Cohen’s κ coefficients [66], we used Stata version 14 [67].^5^

## Results

### Distribution of levels of contact with people with depression

The distribution of participants’ levels of contact with depression is displayed in Table 1. As shown in Table 1, 65.45% of the participants had not watched people with depression in real life (i.e., rank order score < 5), and 80.00% of the participants had no friends or family members with depression (i.e., rank order score < 9). No participants responded with “I have depression” (rank order score = 12)—this result indicates that we successfully recruited undergraduates without clinical depression.

**Table 1:** Distribution of levels of contact with people with depression (*n* = 56)

Rank order score	Item	%	Cumulative %
1	I have never observed a person that I was aware had depression	14.55	14.55
2	I have observed, in passing, a person I believe may have had depression	7.27	21.82
3	I have watched a movie or television show in which a character depicted a person with depression	12.73	34.55
4	I have watched a documentary on the television about depression	30.91	65.45
5	I have observed persons with depression on a frequent basis	10.91	76.36
6	I have worked with a person who had depression at my place of employment	1.82	78.18
7	My job includes providing services to persons with depression	1.82	80.00
8	My job involves providing services/treatment for persons with depression	0.00	80.00
9	A friend of the family has depression	3.64	83.64
10	I have a relative who has depression	10.91	94.55
11	I live with a person who has depression	5.45	100.00
12	I have depression	0.00	100.00

### Numbers of comments and labels obtained from the free description sheet

The participants wrote 608 comments in total on the free description sheets, and we obtained 624 labels from these raw comments. The numbers of raw comments and labels in each domain are summarized in Table 2. A one-way ANOVA showed that the mean numbers of labels differed significantly across the four domains on the free description sheets (*F*(3, 165) = 20.21, *p* < 0.001, $\eta_{p}^{2}$ = 0.27). Post hoc tests with Bonferroni correction showed the following: the mean numbers of labels for *benefits of helping* were significantly larger than those for *costs of helping*, *costs of not helping,* and *benefits of not helping* (*t*s > 2.93, *p*s < 0.030, *d*s > 0.27); the mean number for *costs of helping* was significantly larger than that for *benefits of not helping* (*t*(55) = 6.28, *p* < 0.001, *d* = 0.59); there were no other significant differences (*t*s < 2.30, *p*s > 0.154, *d*s < 0.22).

**Table 2:** Numbers of comments and labels obtained from free description sheets (*n* = 56)

	Number of comments	Number of labels
	*M* (*SD*)	*M* (*SD*)
Costs of helping	2.91 (1.13)	2.95 (1.17)
Benefits of helping	3.39 (1.26)	3.50 (1.29)
Costs of not helping	2.38 (1.21)	2.52 (1.35)
Benefits of not helping	2.18 (0.92)	2.18 (0.92)

### Overview of the category content

The categories obtained in each domain are summarized in Table 3. The labels in each domain were divided into categories of: (i) costs/benefits for the participants themselves, (ii) costs/benefits for the person in the vignette, (iii) negative/positive effects on interpersonal relationships, and (iv) other labels. It can be seen that the distributions of the labels were markedly different among the four domains (e.g., Domain B included 66 labels [33.67%] focusing on interpersonal relationships, whereas Domain D included only 6 labels [4.92%] focusing on them). The four domains had a large number of themes in common: for example, the theme focusing on the distress felt when symptoms got worse was expressed as Category 2 in Domain A and as Category 3 in Domain C. In contrast, some themes were domain-specific: for example, the theme of respecting the autonomy and opportunity of the person in the vignette was observed only in Domain D (Category 5).

**Table 3:** Categories for costs/benefits of helping/not helping the person in the vignette

Domain A: Costs of helping (165 labels)	Domain C: Costs of not helping (141 labels)
Self (135 labels; 81.82%)	Self (87 labels; 61.70%)
1. Psychological distress: Distress felt when conducting helping behavior (53 labels; 32.12%)	1. Psychological distress: Distress felt when making the decision not to help (25 labels; 17.73%)
	2. Psychological distress: Distress over keeping the friendship (40 labels; 28.37%)
2. Psychological distress: Guilt and powerlessness felt when helping behavior failed (10 labels; 6.06%)	3. Psychological distress: Self-reproof felt when symptoms got worse (13 labels; 9.22%)
3. Material expenses (56 labels; 33.94%)	4. Loss of opportunity to gain knowledge and information (9 labels; 6.38%)
4. Loss of energy (16 labels; 9.70%)	
Person in the vignette (6 labels; 3.64%)	Person in the vignette (12 labels; 8.51%)
5. Psychological distress for the person in the vignette (6 labels; 3.64%)	5. Loss of opportunity to improve symptoms (12 labels; 8.51%)
Interpersonal relationships (20 labels; 12.12%)	Interpersonal relationships (37 labels; 26.24%)
6. Deterioration of the friendship (18 labels; 10.91%)	6. Deterioration and dissolution of the friendship (26 labels; 18.44%)
7. Loss of reputation (2 labels; 1.21%)	7. Loss of reputation (11 labels; 7.80%)
Other labels (4 labels; 2.42%)	Other labels (5 labels; 3.55%)
8. Other labels (4 labels; 2.42%)	8. Other labels (5 labels; 3.55%)

Domain B: Benefits of helping (196 labels)	Domain D: Benefits of not helping (122 labels)
Self (87 labels; 44.39%)	Self (101 labels; 82.79%)
1. Psychological benefits (40 labels; 20.41%)	1. Avoidance of psychological distress (63 labels; 51.64%)
2. Gaining knowledge, experience, and skills (32 labels; 16.33%)	2. Gaining the opportunity to think over the possible approaches (2 labels; 1.64%)
3. Resolution or avoidance of psychological distress (15 labels; 7.65%)	
	3. Avoidance of material expenses (36 labels; 29.51%)
Person in the vignette (42 labels; 21.43%)	Person in the vignette (13 labels; 10.66%)
4. Psychological benefits for the person in the vignette (42 labels; 21.43%)	4. Avoidance of psychological distress for the person in the vignette (8 labels; 6.56%)
	5. Respecting autonomy and opportunity (5 labels; 4.10%)
Interpersonal relationships (66 labels; 33.67%)	Interpersonal relationships (6 labels; 4.92%)
5. Maintaining or deepening the friendship (60 labels; 30.61%)	6. Avoiding the risks of deteriorating friendship (6 labels; 4.92%)
6. Gaining a reputation (6 labels; 3.06%)	
Other labels (1 label; 0.51%)	Other labels (2 labels; 1.64%)
7. Other labels (1 label; 0.51%)	7. Other labels (2 labels; 1.64%)

### Reliabilities of the categories and subcategories

As summarized in Table 4, all Cohen’s κ coefficients [66] between two raters indicated almost perfect agreement (κs > 0.80) for the categories, according to the criteria proposed by Landis and Koch [69]. The κ coefficients for the subcategories (detailed later in the Results section) indicated substantial agreement (κs > 0.60) for the *costs of helping* and *costs of not helping* domains, and almost perfect agreement (κs > 0.80) for the *benefits of helping* and *benefits of not helping* domains. Overall, all categories and subcategories obtained in the present study exhibited high reliability.

**Table 4:** Numbers and reliabilities of categories and subcategories for costs/benefits of helping/not helping the person in the vignette

	Categories		Sub-categories	
	Number	Cohen’s κ	Number	Cohen’s κ
Costs of helping	8	0.81	13	0.78
Benefits of helping	7	0.87	21	0.81
Costs of not helping	8	0.82	12	0.79
Benefits of not helping	6	0.93	8	0.84

### Domain A: Costs of helping (including 165 labels)

#### Category 1: Psychological distress: Distress felt when conducting helping behavior (53 labels; 32.12%)

More than one-third of participants wrote about the psychological distress they themselves would experience (Categories 1 and 2). In particular, many participants expected that they would feel uncomfortable or burdened while listening to the person in the vignette. We classified such labels concerning the distress felt when engaging in helping behaviors into Category 1. This category included the following three subcategories: *feeling depressed* (29 labels; e.g., “Maybe I will feel depressed by listening to ‘A’.” [Participant 5]); *stressful and annoying* (15 labels; e.g., “Listening to ‘A’ will be a stressful event for me.” [Participant 48]); and *feeling responsible for the problem of “A”* (9 labels; e.g., Maybe I should take responsibility for all ‘A’s’ problems.” [Participant 27]). In the analyses, we selectively classified labels that vaguely described psychological distress (i.e., did not mention specific feelings such as depressed mood) into the *stressful and annoying* subcategory.

#### Category 2: Psychological distress: Guilt and powerlessness felt when helping behavior failed (10 labels; 6.06%)

Some participants imagined situations in which they engaged in helping behavior but it did not work, and they mentioned the feelings of guilt and powerlessness they felt in such situations (e.g., “I will feel guilty and blame myself if ‘A’ does not get well with my help” [Participant 47]). We classified such labels, including worries about the failure of helping behavior, into Category 2.

#### Category 3: Material expenses (56 labels; 33.94%)

In contrast to Categories 1 and 2 regarding psychological distress, Category 3 concerned specific material expenses that the participants would have to spare. This category included the subcategories of *loss of time* (49 labels; e.g., “I have to spare some time to listen to ‘A’.” [Participant 50]); *loads of learning knowledge and skills* (4 labels; e.g., “I have to learn basic knowledge about depression.” [Participant 20]); and *monetary expenses* (3 labels; e.g., “I have to spend some money if I listen to ‘A’ in cafes or restaurants.” [Participant 33]).

#### Category 4: Loss of energy (16 labels; 9.70%)

Some participants provided labels such as “I will lose some energy” (Participant 49) or “It may be burdensome for me” (Participant 14). Although such labels obviously mentioned costs for the participants themselves, it was unclear whether these labels concerned either psychological distress or material expenses. We classified such unclear labels concerning the costs for the participants into Category 4.

#### Category 5: Psychological distress for the person in the vignette (6 labels; 3.64%)

Although we explained that empathy is not the only determinant of helping behavior in the slideshow and instructed the participants to think about the costs and benefits for themselves, some participants reported feeling psychological distress only for the person in the vignette. We classified such labels (e.g., “‘A’ may feel the helping behavior is burdensome.” [Participant 34]) into Category 5.

#### Category 6: Deterioration of the friendship (18 labels; 10.91%)

A number of labels mainly concerned the possible negative effects of helping behaviors on interpersonal relationships and did not deal with the direct costs for themselves (Categories 6 and 7). We classified the label that concerned the deterioration of friendship into Category 6. This category included subcategories of *deterioration of friendship with the person in the vignette* (12 labels; e.g., “Maybe ‘A’ will feel the helping behavior is annoying, and our friendship will deteriorate.” [Participant 26]) and *being scolded or harmed by the person in the vignette* (6 labels; e.g., “Maybe I will be scolded violently by ‘A’ for interfering him/her.” [Participant 15]).

#### Category 7: Loss of reputation (including 2 labels, 1.21%)

A few labels concerned the negative perceptions of bystanders, rather than the person in the vignette (e.g., “Others may regard me as a hypocrite.” [Participant 29]). We classified such labels involving worries about loss of reputation into Category 7.

#### Category 8: Other labels (including 4 labels, 2.42%)

Some labels did not correctly describe the costs of the helping behavior (e.g., “Maybe I can do something for ‘A’.” [Participant 25]). Such incorrect answers were classified into Category 8 and were treated as “other labels.”

### Domain B: Benefits of helping (including 196 labels)

#### Category 1: Psychological benefits (40 labels; 20.41%)

A variety of psychological benefits for the participants was written into Domain B, and we classified such labels into Category 1. This category included the following six subcategories: *pleasure of helping someone* (10 labels; e.g., “I will be pleased to know that I could serve someone else.” [Participant 1]); *feeling better* (7 labels; e.g., “I will feel better.” [Participant 41]); *getting satisfied* (11 labels; e.g., “I will be satisfied, because I engaged in helping behavior that is socially desirable.” [Participant 10]); *feelings of self*-*efficacy* (6 labels; e.g., “I will gain confidence in myself.” [Participant 15]); *being relieved* (3 labels; e.g., “I will be relieved by the fact that ‘A’ can talk with me.” [Participant 47]); and *gaining the opportunity to reflect on oneself* (3 labels; e.g., “Helping ‘A’ will provide me with an opportunity to reflect on myself.” [Participant 33]).

#### Category 2: Gaining knowledge, experience, and skills (32 labels; 16.33%)

Aside from the psychological benefits described in Category 1, some participants responded with the helping behavior benefits of gaining knowledge, experience, and skills. Such labels were classified into Category 2, and this category included the following four subcategories: *gaining knowledge about depression* (11 labels; e.g., “I am interested in mental health problems including depression, so I would be happy to learn more about depression through the helping behavior.” [Participant 26]); *gaining experience through interacting with people with depression* (9 labels; e.g., “I expect that I can expand my experience by carrying out helping behaviors for people with depression.” [Participant 19]); *gaining skills in helping and expressing empathy* (9 labels; e.g., “I will learn how I should help people with depression in the future.” [Participant 44]); and *gaining self*-*help skills* (3 labels; e.g., “I expect that I can learn how to help myself when I get depressed like ‘A’.” [Participant 36]).

#### Category 3: Resolution or avoidance of psychological distress (15 labels; 7.65%)

In contrast to Categories 1 and 2, which focused on the gains for the participants, Category 3 highlighted the resolution or avoidance of possible psychological distress. This category included the subcategories of *resolving worries and anxiety* (9 labels; e.g., “My worries will be decreased.” [Participant 56]) and *avoiding feelings of guilt and regret* (6 labels; e.g., “I can avoid feeling guilty for abandoning ‘A’.” [Participant 10]).

#### Category 4: Psychological benefits for the person in the vignette (42 labels; 21.43%)

In Domain B, many labels concerned the psychological benefits for the person in the vignette rather than for the participants themselves (cf. few labels for the psychological distress of the person in the vignette were obtained in Domain A). Such labels were classified into Category 4, and this category included the subcategories of *improvement in symptoms of depression* (37 labels; e.g., “I hope that ‘A’ gets well, as he/she used to be.” [Participant 16]) and *preventing worsening the problem* (5 labels; e.g., “Maybe I can prevent suicide.” [Participant 18]).

#### Category 5: Maintaining or deepening the friendship (60 labels; 30.61%)

Compared with Domain A, Domain B included more labels concerning interpersonal relationships (Categories 5 and 6). We classified the labels that included the positive effects of helping behaviors on the friendship with the person in the vignette into Category 5. This category included the following five subcategories: *deepening friendship* (28 labels; e.g., “Maybe I can get closer to ‘A’ as a friend.” [Participant 20]); *maintaining friendship* (12 labels; e.g., “We can stay good friends.” [Participant 6]); *being thanked* (7 labels; e.g., “Maybe I will be thanked by ‘A’.” [Participant 31]); *further understanding of the counterpart* (6 labels; e.g., “I can understand more about ‘A’.” [Participant 7]); and *expected rewards from the counterpart* (7 labels; e.g., “By helping ‘A’, he/she will help me as a reward when I am in trouble.” [Participant 42]).

#### Category 6: Gaining a reputation (6 labels; 3.06%)

Some labels concerned the positive views of bystanders rather than the person in the vignette (e.g., “I will gain a reputation among others by helping ‘A’.” [Participant 50]). We classified such labels concerning the expectation of building a reputation into Category 6.

#### Category 7: Other labels (1 label; 0.51%)

In the analyses, we could not capture the meaning of one label (Participant 33) in Domain B. We classified that label into Category 7 and treated it as an “other label.”

### Domain C: Costs of not helping (including 141 labels)

#### Category 1: Psychological distress: Distress felt when making the decision not to help (25 labels; 17.73%)

Many of the participants wrote about the psychological distress they themselves would experience (Categories 1, 2, and 3). In particular, a number of participants worried that they would feel uncomfortable or guilty when deciding to not help the person in the vignette. We classified such labels into Category 1, which included the subcategories of *senses of guilt* (15 labels; e.g., “I will feel guilty. It is painful for me to betray a friend of mine when I notice his/her problem.” [Participant 4]) and *self*-*hatred* (10 labels; e.g., “I will hate myself for pretending not to notice the problem of ‘A’.” [Participant 17]).

#### Category 2: Psychological distress: Distress over keeping the friendship (40 labels; 28.37%)

A certain number of participants worried that they must keep in touch with the person in the vignette without helping him/her, and that they would feel uncomfortable in such situations. We classified such labels on the worries over the distress of keeping a friendship into Category 2. This category included the subcategories of *continuing worries and anxiety* (21 labels; e.g., “I shall never come to the end of worries about my friend.” [Participant 3]); *getting tired of keeping the friendship* (13 labels; e.g., “I have to keep in touch with ‘A,’ who will stay unwell.” [Participant 43]); and *getting depressed* (6 labels; e.g., “I will also be depressed by worrying about ‘A’ too much.” [Participant 46]). We first classified labels that described the depressed mood of the participants into the *getting depressed* subcategory, and then we classified other labels in Category 2 into either the *continuing worries and anxiety* or *getting tired of keeping friendship* subcategories.

#### Category 3: Psychological distress: Self-reproof felt when symptoms got worse (13 labels; 9.22%)

Some participants imagined situations in which the symptoms of the person in the vignette worsened and mentioned feelings of self-reproof in such situations (e.g., “I will blame myself and regret not helping ‘A’ if he/she undergoes tragic situations afterward” [Participant 29]). We classified such labels into Category 3.

#### Category 4: Loss of opportunity to gain knowledge and information (9 labels; 6.38%)

Aside from the psychological distress described in Categories 1, 2 and 3, some participants worried about losing the opportunity to gain knowledge of depression and detailed information about the person in the vignette. Such labels were classified into Category 4, and this category included the subcategories of *losing the opportunity to gain knowledge about depression* (5 labels; e.g., “I cannot gain practical knowledge about depression.” [Participant 29]) and *losing the opportunity to get detailed information about the person in the vignette* (4 labels; e.g., “I cannot understand the reason why ‘A’ got so unwell.” [Participant 11]).

#### Category 5: Loss of opportunity to improve symptoms (12 labels; 8.51%)

As in Domain A, some participants wrote about the costs for the person in the vignette. These labels mainly concerned loss of opportunity to improve symptoms (e.g., “Maybe symptoms of ‘A’ will get much worse.” [Participant 46]), and we classified such labels into Category 5.

#### Category 6: Deterioration and dissolution of the friendship (26 labels; 18.44%)

A certain number of participant labels mainly concerned the possible negative effects of not helping on interpersonal relationships and did not respond concerning the direct costs to themselves (Categories 6 and 7). We classified the label that concerned the deterioration and dissolution of friendship into Category 6 (e.g., “Maybe our friendship will be dissolved.” [Participant 49]).

#### Category 7: Loss of reputation (11 labels; 7.80%)

Some labels concerned the negative views of bystanders rather than the person in the vignette (e.g., “Others may regard me as a cold-hearted person.” [Participant 25]). We classified such labels on the worries concerning loss of reputation into Category 7.

#### Category 8: Other labels (5 labels; 3.55%)

Some labels described the benefits, rather than the costs, of not helping (e.g., “I can save my energy.” [Participant 9]). Such labels that failed to describe the costs were classified into Category 8 and were treated as “other labels.”

### Domain D: Benefits of not helping (including 122 labels)

#### Category 1: Avoidance of psychological distress (63 labels; 51.64%)

In Domain D, most participants wrote about the direct benefits to themselves (Categories 1, 2, and 3). In particular, more than half of the labels in Domain D concerned the avoidance of psychological distress. We classified such labels into Category 1, and it included the subcategories of *avoidance of responsibility* (42 labels; e.g., “I need not undertake others’ problems and worries.” [Participant 10]), and *avoidance of depressed mood and anxiety* (21 labels; e.g., “By not listening to ‘A’, I will neither get depressed nor feel anxious.” [Participant 5]).

#### Category 2: Gaining the opportunity to think over the possible approaches (2 labels; 1.64%)

A few participants wrote that they would gain the opportunity to think over the possible approaches to effectively help the person (e.g., “I can think of alternative approaches to help ‘A’ instead of just listening to ‘A’.” [Participant 34]). We classified such labels into Category 2.

#### Category 3: Avoidance of material expenses (36 labels; 29.51%)

A number of labels concerned the avoidance of material expenses, and most of them focused on the avoidance of loss of time (e.g., “I can save time and spend it on myself.” [Participant 45]). We classified such labels into Category 3.

#### Category 4: Avoidance of psychological distress for the person in the vignette (8 labels; 6.56%)

Some labels concerned the psychological benefits for the person in the vignette rather than for the participants themselves (Categories 4 and 5). We classified the labels that viewed avoidance of psychological distress as a benefit for the person in the vignette into Category 4 (e.g., “By being left alone, ‘A’ will not have to feel beholden to others.” [Participant 8]).

#### Category 5: Respecting autonomy and opportunity (5 labels; 4.10%)

Some participants responded concerning the importance of respecting the autonomy and opportunity of the person in the vignette. Such labels were classified into Category 5, which included the subcategories of *respecting the autonomy of the person in the vignette* (2 labels; e.g., “Maybe ‘A’ will realize on his/her own that he/she should see a doctor.” [Participant 1]) and *expecting help from other bystanders* (3 labels; e.g., “Perhaps ‘A’ will be helped by someone else who is better at helping behaviors than I.” [Participant 15]).

#### Category 6: Avoiding the risks of deteriorating friendship (6 labels; 4.92%)

Compared with Domains A, B, and C, fewer labels concerning interpersonal relationships were in Domain D. These labels focused on the positive effects of not helping the friendship with the person in the vignette (e.g., “I can avoid changing the relationship between ‘A’ and me.” [Participant 20]), rather than on their reputation as seen by the bystanders. We classified these into Category 6.

#### Category 7: Other labels (2 labels; 1.64%)

A few labels described the costs, rather than the benefits, of not helping (e.g., “I will feel embarrassed and uncomfortable.” [Participant 25]). Such labels that failed to describe the benefits were classified into Category 7 and were treated as “other labels.”

## Discussion

The present study primarily aimed to explore and list what kinds of costs and benefits are perceived among a sample of Japanese undergraduates regarding first aid for peers with depression. We obtained free descriptive comments from the participants on the perceived costs/benefits of helping/not helping, statistically compared the numbers of labels across 2 (costs/benefits) × 2 (helping/not helping) domains, and then listed the content of these labels by conducting qualitative content analysis in a stepwise manner.

### Discussion of the supplemental quantitative comparisons of labels

The supplemental quantitative comparisons of numbers of labels across domains provide some empirical evidence that the economic perspective on helping behaviors [41–43] should be considered in research on MHFA for youth with depression. As shown in Table 1, the participants perceived a certain number of *costs of helping* and *benefits of not helping,* which work as suppressors of helping behavior. Although these suppressors were less frequently perceived than were the *benefits of helping* (i.e., motivators of helping behavior), ANOVAs showed that mean numbers of labels for the *costs of helping* and *benefits of not helping* (i.e., suppressors) domains were not significantly different from those for the *costs of not helping* (i.e., motivators) domain. In contrast to the previous research on the YMHFA program that have focused on mental health literacy and have implicitly assumed human altruism, these results reveal that people perceive both motivators and suppressors in making decisions to help youth with depression. It is also noteworthy that these results were obtained from a sample of undergraduates majoring in psychology, considering that even those participants who were expected to have an interest in depression perceived a certain number of suppressors in extending helping behaviors. It is possible that lay people who do not know much about psychological problems perceive many more suppressors when deciding to help youth with depression.

### Discussion of the qualitative analyses concerning the perceived costs/benefits of helping/not helping

The present study mainly aimed to explore the determinants, other than empathy, of helping behaviors, and we obtained a variety of categories regarding the costs/benefits for the participants themselves by conducting qualitative analyses (see Table 3). The results obtained for Domains A (*costs of helping*) and B (*benefits of helping*) suggest that helping behavior can evoke psychological distress (e.g., be stressful and annoying) as well as simultaneous psychological benefits (e.g., elicit the pleasure of helping someone). These conflicts between the costs and benefits seem to be quite complicated, because costs and benefits involved in helping behaviors include features other than psychological ones, such as *material expenses*, *loss of energy* (Categories 3 and 4 in Domain A), and *gains of knowledge, experience, and skills* (Category 2 in Domain B).The categories in Domains A and B also indicate that people perceive conflicts between costs and benefits because they cannot foresee the outcomes of the helping behavior. As indicated by Categories 1 and 3 in Domain B (see Table 4), people are likely to have positive feelings and avoid psychological distress if the helping behavior results in success; on the other hand, as indicated by Category 2 in Domain A (see Table 4), they will probably suffer feelings of guilt and powerlessness if the helping behavior results in failure.

The results obtained in Domains C (*costs of not helping*) and D (*benefits of not helping*) indicate similar ideas. The categories in these domains suggest that the decision to not help peers with depression can evoke psychological distress, as described in Category 1 in Domain C (e.g., sense of guilt, self-hatred). It can also lead to the avoidance of other kinds of psychological distress as described in Category 1 in Domain D (e.g., feelings of responsibility, anxiety). Conflicts between the costs and benefits of not helping seem to be quite complicated, because they include features other than psychological distress and benefits, such as *loss of opportunity to gain knowledge and information* (Category 4 in Domain C), *gaining the opportunity to think over possible approaches,* and *avoidance of material expenses* (Categories 2 and 3 in Domain D). Categories 2 and 3 in Domain C also indicate that people have to suffer a variety of psychological distress because they cannot foresee the results of the decision to not help. As indicated by Category 2, they are likely to experience continued worries and anxiety when their peers cannot improve their symptoms on their own. Moreover, as indicated by Category 3, they have to suffer feelings of self-reproof when their peers’ symptoms of depression worsen.

In sum, those categories regarding the costs/benefits for the participants themselves indicate that young people have to undergo conflicts regardless of whether they decide to help their peers with depression. These results vividly depict the content of conflicts regarding the decision of whether to help peers with depression and indicate the difficulty of the decision-making. Considering that the choice to either help or not is accompanied by conflicts in the costs and benefits for the help providers, it seems that the accommodation of perceived costs and benefits is greatly needed to motivate young people to conduct first aid for their peers with depression.

Interestingly, although we aimed to explore the determinants of helping behavior other than empathy, every domain included categories that concerned the costs/benefits for the person in the vignette rather than for the participants themselves (see Table 4). In particular, the majority (21.43%; Category 4) of the labels in Domain B (*benefits of helping*) concerned benefits of helping behaviors for the person in the vignette. These results possibly reflect participants’ expectancy of the reciprocity of helping behaviors. As discussed in studies of evolutionary and experimental psychology [e.g., [70–72] ], individuals who tend to help others are likely to be helped in return when they have some trouble; as a result, they are likely to be more adaptive. This expectancy of reciprocity is considered to be shared by most cultural groups of the world [73] and forms the societal norms of reciprocity [74]. It is therefore plausible that the participants were aware of the norms of reciprocity, assumed that helping their peers should benefit them in return, and then frequently responded concerning the benefits of helping their peers.

It is also noteworthy that every domain included categories concerning the negative/positive effects on interpersonal relationships (i.e., friendship or reputation from others) rather than on specific individuals (i.e., the participants themselves or the person in the vignette). In particular, such labels occupied 33.67% (Categories 5 and 6) of all labels obtained in Domain B (*benefits of helping*). These results may reflect participants’ desires for belongingness (i.e., relatedness), which have been regarded as core motivations of human behavior in previous evolutionary and social psychological studies [75–77]. These studies have discussed that cooperation with others serves as an adaptive strategy for most individuals; thus, they hypothesized that belongingness is indispensable for humans to survive. This belongingness hypothesis has been supported by plenty of empirical findings (e.g., [78–80]), and we believe that the content of the categories on the negative/positive effects on interpersonal relationships obtained in the present study are consistent with this hypothesis. These results are insightful because they reveal that not only the direct costs/benefits for help providers and receivers but also the negative/positive effects on interpersonal relationships can be determinants of helping behavior. In contrast to the existing YMHFA program, which mainly focused on the thoughts and feelings of help providers and receivers, these results tell us that we have to consider whether a behavior is likely to be accepted within a specific social context to determine whether we should decide to help our peers with depression.

### Limitations and future directions

Several limitations of the present study should be noted here, along with some directions for future research. First, the cross-cultural applicability of the present findings is unclear. As previous studies of cross-cultural psychology have discussed, East Asian countries, including Japan, tend to have collectivistic cultures. This is in direct contrast to North American countries, which tend to have individualistic cultures [64, 81–83]. These studies have also shown that East Asians tend to have interdependent self-construal; in other words, they define who they are with reference to social contexts and interpersonal relationships. It is therefore possible that young people in countries outside East Asia do not care so much about the negative/positive effects of helping behavior on interpersonal relationships. It is also noteworthy that East Asians tend to view persons who express negative feelings as weak, shameful, and disturbing of social harmony [84]. Considering also that the Japanese have cross-culturally strong stigmas toward mental disorders, including depression [53], it is possible that the Japanese perceive more suppressors (i.e., *costs of helping* and *benefits of not helping*) in helping their peers with depression. Therefore, future research should examine cross-cultural differences in the amount and content of the perceived costs and benefits regarding first aid for youth with depression.

Second, most of the participants in the present study had limited real-life interpersonal contact with people with depression (see Table 1). It is therefore unclear to what extent the present findings obtained in the vignette-based design reflect the costs and benefits that young people perceive when they see their peers with depression in real-life situations.^6^ Future research should, therefore, selectively recruit young people who had interpersonal contact with people with depression by contacting family associations and so forth, and they should examine how much they agree with the present findings.

Third, we cannot clearly determine which kind of costs and benefits are most crucial for young people to determine if they should or should not help their peers with depression. Although we can examine the numbers of labels classified into each category of perceived costs/benefits by using the present data, these numbers possibly reflect relative availability rather than the importance of categories. Another quantitative examination is therefore needed in the future to more closely estimate the relative importance of perceived costs and benefits. One solution is to conduct a prototype analysis, which is a mixed method of qualitative and quantitative analyses to identify a core component of a specific psychological construct [85–90]. Another possible solution is to measure how much people perceive the kinds of costs and benefits by using Likert scales and examine how they predict the amount of helping behaviors or behavioral intentions, neither of which were measured in the present study. It would also be beneficial for the field to compare the magnitude of predictability of helping behavior between altruism and perceived costs/benefits.

Fourth, because the present study prioritized listing the contents of perceived costs/benefits and helping/not helping, and collected comments on the four domains separately, we could not capture the rich context behind those comments or conflict across those domains. Future research, therefore, should conduct in-depth qualitative interviews on those who had contact with peers with depression to explore such context and conflicts. Such explorations should help discuss how future YMHFA programs could resolve participants’ conflicting emotions and motivate them to conduct helping behavior in life.

Fifth, since we selectively focused on the helping behaviors provided by young people toward their peers with depression, the generalizability of the present findings within the MHFA research context remains unclear. Future research should therefore explore the content of perceived costs and benefits regarding MHFA within broader contexts, including mental disorders other than depression and older age groups. Such explorations will enable us to design tailored educational content to motivate the public to conduct various types of MHFA.

## Conclusion

To the best of our knowledge, this is the first study to empirically examine perceived costs and benefits among youth regarding helping behavior toward their peers with depression. The present qualitative study provided implications for future YMHFA programs to motivate participants to conduct helping behavior. The quantitative comparison of numbers of labels on costs/benefits and helping/not helping, which was conducted as a supplementary analysis, indicate that young people perceive suppressors—the components that have been overlooked in the YMHFA program that have implicitly assumed human altruism—as well as motivators in helping behavior. The qualitative content analysis of costs/benefits and helping/not helping, which was conducted as a main analysis, successfully listed contents of these costs and benefits. The list covered a wide range of themes, conveying that the YMHFA should focus the negative/positive effects of helping behavior not only on the receivers of help but also on providers and interpersonal relationships (see Table 3). They also hold numerous implications for future research to explore how to resolve conflicts of the costs and benefits to motivate young people to conduct helping behaviors (see the limitations and future directions subsection for further details). The present study, therefore, contributed to the existing YMHFA program by providing a first theoretical step in the development of educational content aimed at promoting public helping behaviors in realistic situations.

## Supplementary information

**Additional file 1.** Slideshow.

**Additional file 2.** Vignette.

**Additional file 3.** Free description sheet.

## Data Availability

The datasets used and/or analysed during the current study are available from the corresponding author on reasonable request.

## Abbreviations

ANOVA: Analysis of variance
DSM: Diagnostic and Statistical Manual of Mental Disorders
MHFA: Mental Health First Aid
YMHFA: Youth Mental Health First Aid

## Footnotes

1 These students had completed mandatory psychology courses, which include lectures of knowledge about depression and other mental disorders, in the first of two semesters in 2017. Therefore, we supposed that these students would know about depression when we recruited participants during the second semester.

2 We divided the participants into small groups and conducted the same surveys to prevent satisficing (i.e., answering questionnaires carelessly to save cognitive effort), which frequently occurs when conducting paper-and-pencil surveys with a large number of participants simultaneously and collecting data online [54, 55]. No interactive tasks between participants such as group discussions were conducted in the present survey.

3 Because age differences are frequently emphasized in Japanese interdependent and hierarchical society [63, 64], the participants’ responses could be biased if we reminded participants of age differences between them and “A” in the vignette. Therefore, we unspecified the age of “A” and explained that “A” is a good university friend of the readers, to imply that the participants and “A” are connected not by a hierarchical junior-senior relationship but by friendship.

4 We assumed that some participants would give a few answers and finish the task in a few minutes to save cognitive effort unless we set a time restriction on the task. Therefore, we set the 12-minute time restriction to indicate an intensive commitment to the task was required. No participants were still writing their answers at the 12-minute mark.

5 Because the present study primarily aimed to explore the content of the perceived costs and benefits of first aid using qualitative analyses, we did not conduct any a priori power calculations regarding the supplemental quantitative comparison of the numbers of labels. An ad hoc sensitivity analysis (α = 0.05, 1 − β = 0.80) performed by G*Power version 3.1 [68] showed that the present sample size (*N* = 56) was large enough to detect the small effect size (i.e., $\eta_{p}^{2}$ = 0.024); therefore, we concluded that it is safe to draw conclusions from the results of null-hypothesis statistical tests conducted in the present study.

6 We avoided conducting a sensitivity analysis that compares the participants with/without real-life contact with people with depression here, considering that an ad hoc sensitivity analysis with a limited sample size (i.e., total *N* = 56) would bias the results.
