# Supplementary material for: Exploring perceived costs and benefits of first aid for youth with depression: a qualitative study of Japanese undergraduates
Source: Int J Ment Health Syst. 2020 May 24;14:34. doi: 10.1186/s13033-020-00366-7 (PMC7247138; doi:10.1186/s13033-020-00366-7)
Supplement: Supplementary file 1 — Additional file 1. Slideshow. [file 13033_2020_366_MOESM1_ESM.pdf]

What if your friend develops depression? "Cost and benefit" of helping behavior

Produced by Jun Kashihara[1, 2]
and Shinji Sakamoto[1]
[1] Nihon University
[2] Japan Society for the Promotion of Science

Objective and structure of this educational material

- **Objective: Introducing the following open-ended questionnaire**
  - The questionnaire asks you about “cost and benefit” of “providing/not providing helping behavior” to a friend with depression
- **Structure**
  1. **Explanation of prerequisite knowledge**
     “Cost and benefit” of helping behavior
  2. **Hypothetical case**
     Your friend “A” with depression

1. Explanation of prerequisite knowledge

“Cost and benefit” of helping behavior

Objective of this section

- **Learn the theory of helping behavior as prerequisite knowledge**
  - What is the mechanism that leads to helping behavior?
  - What are “cost and benefit” of helping behavior?

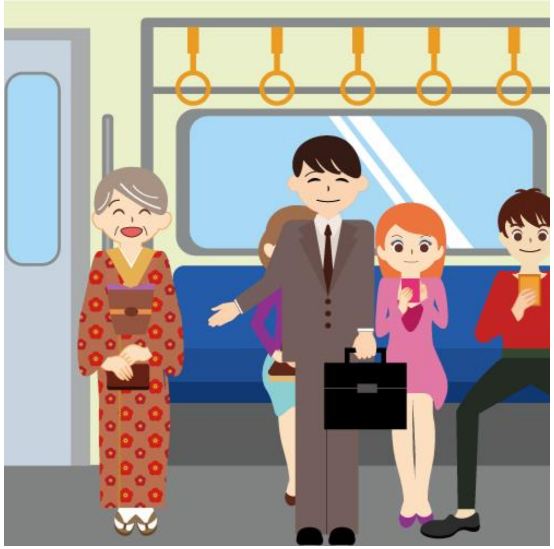

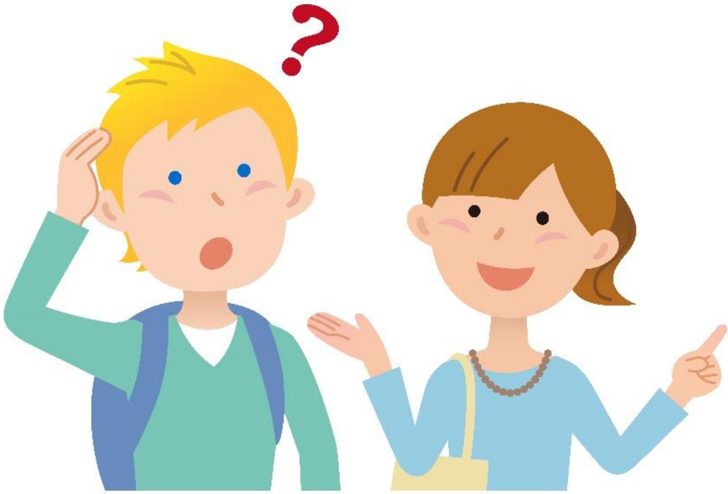

Difference between “Awareness of someone needing help” and “actually helping”

- **The first step of helping behavior is to notice that “helping behavior is needed”**
  - There is someone in need
  - It appears too difficult to solve it on his/her own

- **Even if you notice that “helping behavior is needed”, it may not lead to “actually conduct of helping behavior”**
  - Various factors affect the decision of “helping/not helping”

Factors that might affect the decision

- **Relationship and closeness with the person needing help**
  - Is it a close friend or a stranger?

- **Presence of a bystanders**
  - What if there are many people present?

- **“Cost and benefit” of helping behavior**

  *More details on the next slide

"Cost and benefit" of helping behavior

- **Both options of "helping/not helping" have "cost and benefit" for providers**
  - Providers cannot decide to offer helping behavior for "their benefit" only
- **Providers have to estimate various results before choosing to "help/not help"**

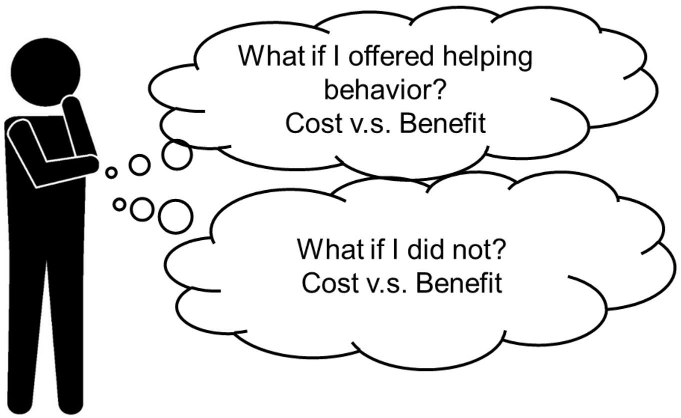

2. Hypothetical case

Your friend “A” with depression

Objective of this section

- **Imagine “what happens if your friend develops depression”**
  - Let’s see a hypothetical case of “A” who got depressed

***Assume that “A” is the same gender as you**

Your friend “A” with depression

“A” is a good friend of yours at the university. You and “A” see each other often both during and outside of classes.

“A” seems to be feeling unusually sad and miserable for the last few weeks. “A” sighs all the time and always gives off gloomy vibes.

“A” is always tired, and has dark circles under his/her eyes from lack of sleep. His/her hair that used to be nice is now messy. With loss of appetite, “A” has lost weight and looks pale.

Your friend “A” with depression (cont.)

During classes, “A” loses concentration. When teachers ask him/her some questions, “A” remains quiet and is unable to answer. It seems that even day-to-day task are too much for him/her.

You see “A” at the university and have noticed that he/she always seems unwell these days.

You thought “‘A’ may be suffering from depression” and “it seems difficult to get out of it on his/her own”.

And you came to think that “someone should take time and listen to ‘A’”.

Additional information on the case

- "A" actually satisfies the diagnostic criteria for depression
- "Taking one's time and listen" is a helping behavior recommended by psychiatrists
  - It can lead to a visit to hospitals

Let us now move onto the questionnaire!
Please open the last page
