# Supplementary material for: Exploring perceived costs and benefits of first aid for youth with depression: a qualitative study of Japanese undergraduates
Source: Int J Ment Health Syst. 2020 May 24;14:34. doi: 10.1186/s13033-020-00366-7 (PMC7247138; doi:10.1186/s13033-020-00366-7)
Supplement: Supplementary file 2 — Additional file 2. Vignette. [file 13033_2020_366_MOESM2_ESM.docx]

**Vignette: Your friend “A” with depression**

“A” is a good friend of yours at the university. You and “A” see each other often both during and outside of classes. “A” seems to be feeling unusually sad and miserable for the last few weeks. “A” sighs all the time and always gives off gloomy vibes. “A” is always tired, and has dark circles under his/her eyes from lack of sleep. His/her hair that used to be nice is now messy. With loss of appetite, “A” has lost weight and looks pale. During classes, “A” loses concentration. When teachers ask him/her some questions, “A” remains quiet and is unable to answer. It seems that even day-to-day task are too much for him/her.

You see “A” at the university and have noticed that he/she always seems unwell these days. You thought “‘A’ may be suffering from depression” and “it seems difficult to get out of it on his/her own”. And you came to think that “someone should take time and listen to ‘A’”.
