# Supplementary material for: Exploring perceived costs and benefits of first aid for youth with depression: a qualitative study of Japanese undergraduates
Source: Int J Ment Health Syst. 2020 May 24;14:34. doi: 10.1186/s13033-020-00366-7 (PMC7247138; doi:10.1186/s13033-020-00366-7)
Supplement: Supplementary file 3 — Additional file 3. Free description sheet. [file 13033_2020_366_MOESM3_ESM.docx]

|  | **Listening to “A”** | **Not listening to “A”** |
| --- | --- | --- |
| **Benefits of...** | **・**  **・**  **・**  **・**  **・**  **・** | **・**  **・**  **・**  **・**  **・**  **・** |
| **Costs of...** | **・**  **・**  **・**  **・**  **・**  **・** | **・**  **・**  **・**  **・**  **・**  **・** |

Participant’s ID
